# Supplementary material for: Broadband thermal imaging using meta-optics
Source: Nat Commun. 2024 Feb 23;15:1662. doi: 10.1038/s41467-024-45904-w (PMC10891089; doi:10.1038/s41467-024-45904-w)
Supplement: Supplementary file 1 — Supplementary Information [file 41467_2024_45904_MOESM1_ESM.pdf]

Supplementary Materials: Broadband Thermal Imaging using Meta-Optics

Luocheng Huang[1], Zheyi Han[1], Anna Wirth-Singh[2], Vishwanath Saragadam[3], Saswata Mukherjee[1], Johannes E. Fröch[1,2], Quentin A. A. Tanguy[1], Joshua Rollag[4,5], Ricky Gibson[5], Joshua R. Hendrickson[5], Phillip W.C. Hon[6], Orrin Kigner[6], Zachary Coppens[7], Karl F. Böhringer[1,8], Ashok Veeraraghavan[3] and Arka Majumdar[1,2*]

[1]Department of Electrical and Computer Engineering, University of Washington, Seattle, WA, USA.
[2]Department of Physics, University of Washington, Seattle, WA, USA.
[3]Department of Electrical Engineering, Rice University, Houston, TX, USA.
[4]KBR, Inc., 3725 Pentagon Blvd., Suite 210 Beavercreek, OH, USA.
[5]Sensors Directorate, Air Force Research Laboratory, 2241 Avionics Circle, Wright-Patterson AFB, OH, USA.
[6]NG Next, Northrop Grumman Corporation, 1 Space Park Drive, Redondo Beach, CA, USA.
[7]CFD Research Corporation, Huntsville, AL, USA.
[8]Institute for Nano-Engineered Systems, University of Washington, Seattle, WA, USA.

*Corresponding author(s). E-mail(s): arka@uw.edu;

Contents

S1 Alignment Sensitivity **3**

S2 PSF Characterization Results **5**

S3 PSF Characterization Setup **8**

S4 Additional Imaging Results Under Ambient Illumination. **9**

S5 Quantitative Image Analysis **11**

S6 Meta-atom Modeling **12**

S7 Fabrication Schematic **14**

S1 Alignment Sensitivity

To study the robustness of the optimized optics with respect to the shift in the sensor plane, we conducted simulations with varying sensor distances. **Fig. S1** presents the spectral Strehl ratio for our complex meta-optics at sensor distances of 10.0 mm, 10.5 mm, and 11.0 mm. The wavelength-averaged Strehl ratios of these simulated systems are 0.0277, 0.0230, and 0.0157. It is observed that the peak of the Strehl ratio - denoting the system's highest performance - shifts towards shorter wavelengths, with an increase in the focal length. This phenomenon is governed by the principle that the wavelength of light and the focal length are inversely proportional in a meta-optics [1]. We find that even though the wavelength-averaged performance is robust against shift in the sensor plane, the individual peaks are still susceptible to the shift.

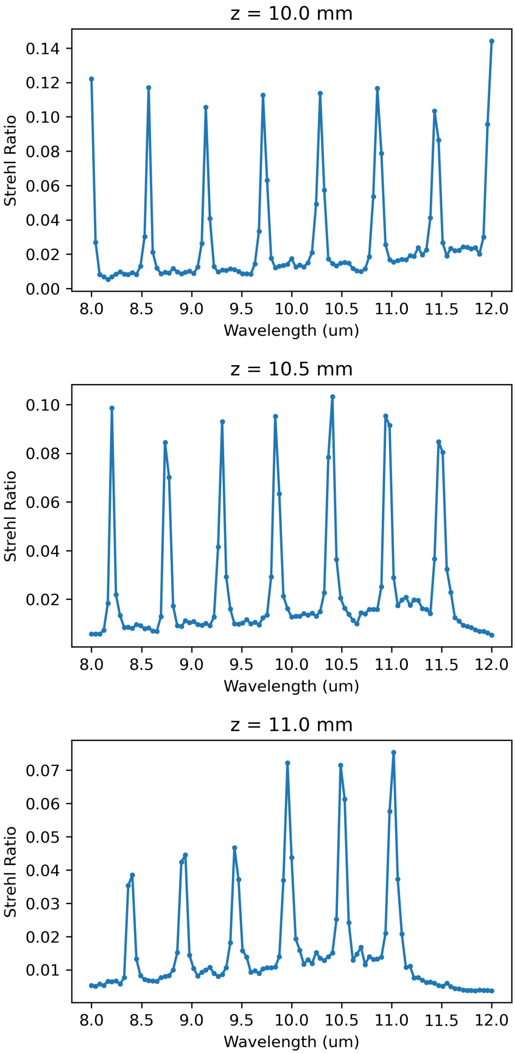

Fig. S1 The Strehl ratio of the complex meta-optic as a function of the input wavelength at different focal planes. From top to bottom, the focal lengths are 10.0, 10.5, and 11.0 mm.

S2 PSF Characterization Results

Our initial experimental step involved measuring the point spread functions (PSFs) of the fabricated devices across various wavelengths, which can be seen in **Fig. S2**. These measurements were taken in light of the alignment sensitivity explained in Section S1, necessitating sparse sampling of PSFs across the spectrum at specific wavelengths—namely, 8.0, 8.5, 9.5, 10.0, and 10.5 micrometers. This selection of wavelengths was chosen to facilitate a direct comparison with simulated PSFs, thereby enabling us to validate our models effectively. It is important to note that due to the aforementioned alignment sensitivity (as discussed in Section S1), we deliberately refrained from capturing PSFs at the optimized wavelengths. Subsequently, the experimentally obtained PSFs were evaluated in conjunction with their corresponding modulation transfer functions (MTFs) and compared against their simulated counterparts. The experimental results displayed a strong correlation with the simulations, underlining the accuracy and reliability of our computational models. The Strehl Ratios computed from the experimental and simulated PSFs are displayed on **Table 1** and **Table 2**.

The hyperboloid metalens performs well at only a single wavelength at 10 μm, with an extremely narrow PSF spot size and a cutoff frequency in the MTF curve of $\sim$ 20 lp/mm, compared to a diffraction limited cutoff frequency of 100 lp/mm. Although we note that the experimental setup incurs additional aberrations from the relay optics effectively decreasing the cutoff frequency. The beam size of the hyperboloid metalens quickly broadens as the operating wavelength deviates from 10 μm showing a cutoff frequency less than 2 lp/mm for other wavelengths. This is due to the inherent chromatic aberration of the diffractive nature of the metalens [2]. In contrast, both MTF-engineered meta-optics maintain a reasonably sized focal spot throughout the entire spectrum and maintain a cutoff frequency over 5 lp/mm for all wavelengths. We emphasize this is exactly what we intended in our design: rather than optimizing for one wavelength, we wanted to have a uniform MTF over the whole wavelength range.

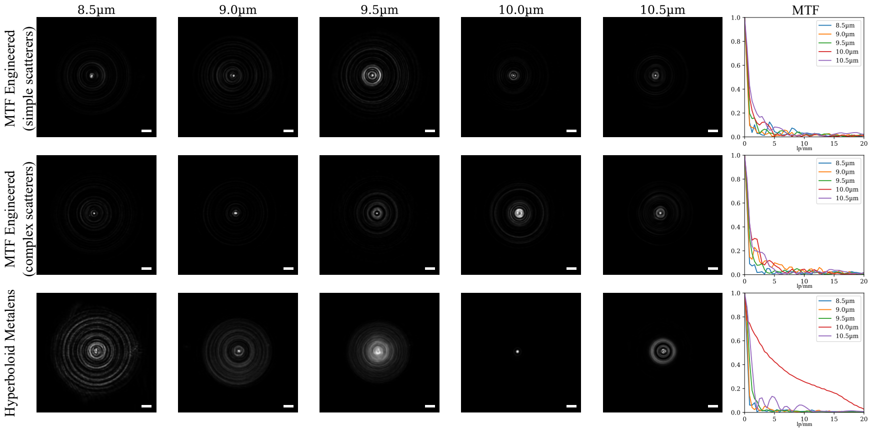

Fig. S2 PSF and MTF characterizations for the hyperboloid metalens (top), MTF engineered meta-optic with simple scatterers (middle), and MTF engineered meta-optic with complex scatterers (bottom). The scale bar is 200 μm in width.

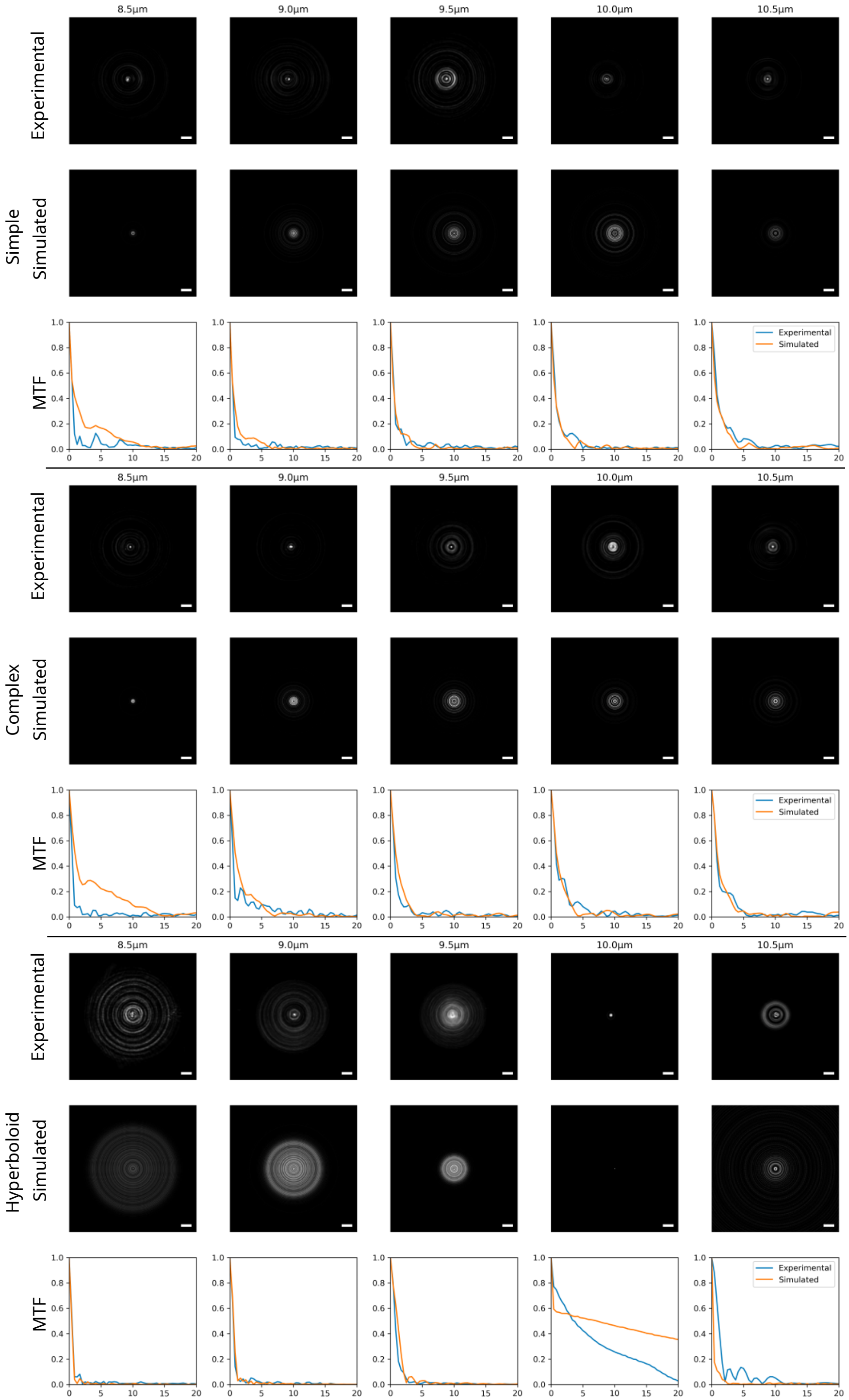

Fig. S3 Simulated vs. experimental PSFs and MTFs. Comparing between "simple", "complex", and hyperboloid meta-optics, from top to bottom, respectively. The scale bar is 200 μm in width.

Table 1 Strehl Ratios from the Experimental PSFs

	8.5 µm	9.0 µm	9.5 µm	10.0 µm	10.5 µm
Simple	0.036	0.03	0.044	0.071	0.076
Complex	0.033	0.063	0.042	0.056	0.063
Hyperboloid	0.02	0.018	0.019	0.366	0.070

Table 2 Strehl Ratios from the Simulated PSFs

	8.5 µm	9.0 µm	9.5 µm	10.0 µm	10.5 µm
Simple	0.072	0.027	0.029	0.038	0.046
Complex	0.069	0.041	0.043	0.06	0.053
Hyperboloid	0.005	0.008	0.014	0.762	0.013

S3 PSF Characterization Setup

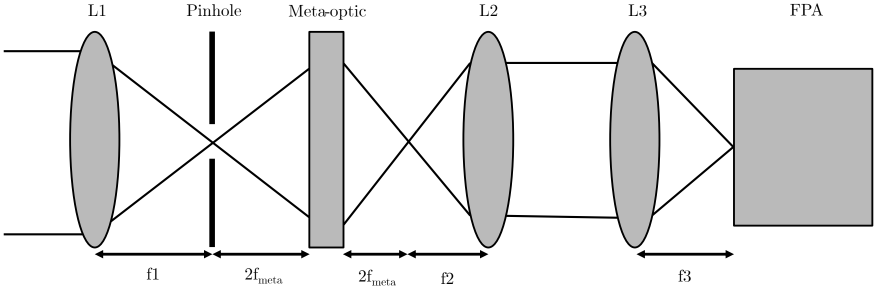

Fig. S4 Schematic of the setup used for PSF characterization of our meta-optics.

A schematic of the setup is shown on **Fig.** S4.

- Laser Source: 8.23 - 10.93 µm QCL
- L1: Ge Aspheric Lens
- Dia. = 25.4 mm, FL f1 = 20.0 mm, NA = 0.63
- Pinhole: Dia. = 30 µm
- Meta: Dia. = 10 mm, FL f_{meta} = 10 mm
- L2: Ge Aspheric Lens
- Dia. = 25.4 mm, FL f2 = 15.0 mm, NA = 0.83
- L3: ZnSe Plano-convex Lens
- Dia. = 25.4 mm, FL f3 = 50.8 mm
- Camera: 15 µm pixel pitch and 640×512 pixels
- Power through pinhole: 30%

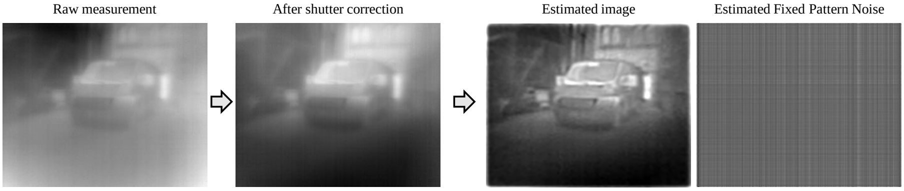

Fig. S5 Deconvolution pipeline. We used an external shutter (flat black body) to remove any internal reflections. After shutter correction, we solved an inverse problem to simultaneously estimate a sharp image, and a fixed pattern noise.

S4 Additional Imaging Results Under Ambient Illumination.

In the main text, we showcase an iterative deep image prior-based algorithm as our image processing backend, as shown in **Fig.** S5. Although this method produces the highest quality thermal images, we demonstrate that our MTF-engineered LWIR meta-optics outperform the hyperboloid metalens, even with a simpler Wiener deconvolution image processing backend. We conducted further imaging tests "in the wild" under ambient daylight conditions, as depicted in **Fig.** S6. We conducted further imaging testing "in the wild" under ambient daylight conditions, shown on **Fig.** S6. These imaging results were performed using Boson 640 camera. The MTF-engineered and forward-designed meta-optics were tested against a refractive lens as the ground truth. First, we imaged a vehicle parked outdoors shown on **Fig.** S6(a). In this scene, the vehicle is clearly visible using the MTF engineered meta-optics with good details on the subject as well as the background. The hyperboloid metalens, however, does not capture the background with any fidelity, also leaving parts of the vehicle without contrast. The second series of captures on **Fig.** S6(b) show a scene of a water fountain in front of building. This series again demonstrates the increase in details via the MTF engineered meta-optic capture, allowing observers to see the trees behind the water fountain, which the hyperboloid metalens fails to demonstrate. The third series on **Fig.** S6(c) shows a woman resting against a window. The hyperboloid metalens fails to capture the subject's glasses, which shows up on the MTF engineered case. Finally, we captured a metal "W" sculpture against vegitations in the background shown on **Fig.** S6(d). The MTF engineered capture displays better subject seperation against the background when compared to the hyperboloid metalens.

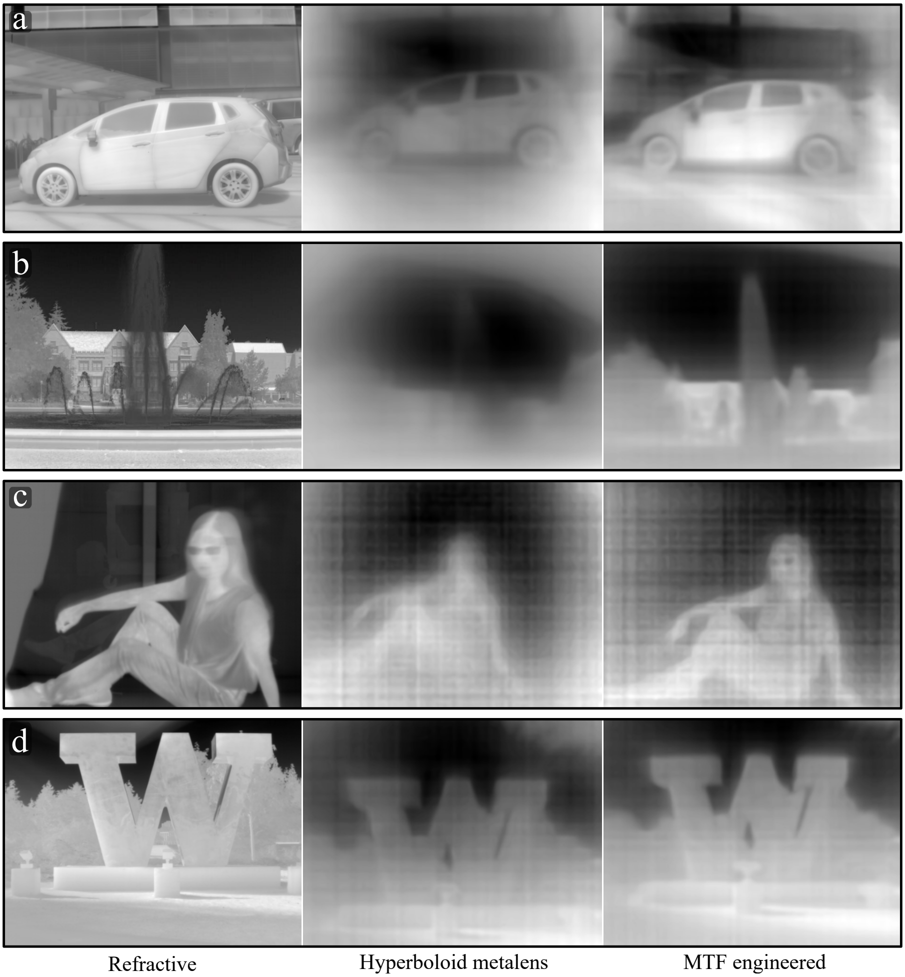

Fig. S6 LWIR imaging “in the wild”. Four scenes are imaged using the refractive LWIR lens (left), the hyperboloid metalens (middle), and the MTF engineered meta-optic with complex scatterers (right). The four scenes were all captured during daytime on a sunny day depicting (a) a parked car on a sunny day; (b) a fountain in the foreground aginst a building in the background; (c) a woman resting against a window; (d) a metallic structure.

S5 Quantitative Image Analysis

To quantify the image quality, we calculate peak signal-to-noise ratios (PSNR) between the ground truth and the broadband imaging captures shown on **Fig. 3** in the main text. Each image is registered using affine transformation to eliminate potential misalignment in the captures. We present the PSNR values for the hyperboloid metalens, MTF engineered with simple scatterers, and with complex scatterers. For each device, we include three different imaging conditions – without a filter, with a $10 \pm 0.25\,\mu m$ bandpass filter, and with a $12 \pm 0.25\,\mu m$ bandpass filter. The PSNR values are shown on **Table 3**.

Table 3 Peak Signal-to-Noise Ratio in dB

	Hyperboloid	Simple	Complex
Broadband	11.49	10.84	13.33
Narrow-band 10 μm	10.74	9.38	10.67
Narrow-band 12 μm	8.26	10.02	12.65

S6 Meta-atom Modeling

Fig. S7 presents the training loss evolution for our simple and complex meta-optic designs over multiple epochs. The models were trained using a 10-layer fully connected network (FCC) with 128 units per hidden layer and a ReLU activation function. Both models were trained using the Adam optimizer, with a learning rate set at 0.0006. The simple meta-optic design concludes training with a remarkably low loss of 0.007, while the more complex design, despite its additional intricacies, achieves a acceptable final loss of 0.048. The consistent decrease in training loss over time, seen in both scenarios, validates the robustness and effectiveness of our deep-learning assisted differentiable framework in managing the design complexities inherent in meta-optics.

A comprehensive analysis to assess the robustness of the scatterers was conducted, emphasizing on the modifications in the correlation coefficient R^2 as perturbations were introduced to the design parameters (p_0, p_1, p_2), as illustrated in **Fig. S8**. This exploration reveals that the scatterers simulated under such perturbations, which mimic deviations during the fabrication process, retain substantial robustness. Specifically, when all three design parameters were simultaneously perturbed, with a standard deviation of $100\mu m$, the correlation coefficient of the predicted phase modulation of the scatterers declined from 0.98 in the unperturbed state to 0.85. Simultaneously, the correlation coefficient for transmission was reduced from 0.93 to 0.71. We highlight that these perturbations are congruent with the Strehl Ratios depicted in Fig. 1 (d). We also attached the simulated ideal Strehl Ratios for the different designs on **Fig. S9**, where no perturbation is introduced.

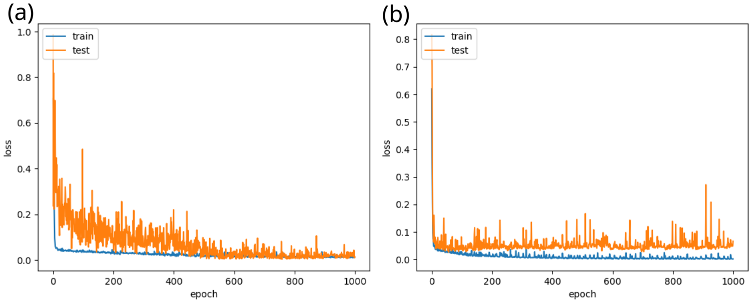

Fig. S7 Meta-optic training loss progress for the simple (a) and the complex (b) meta atoms: each plot shows the downward trend of training loss as a function of epochs, indicating model optimization.

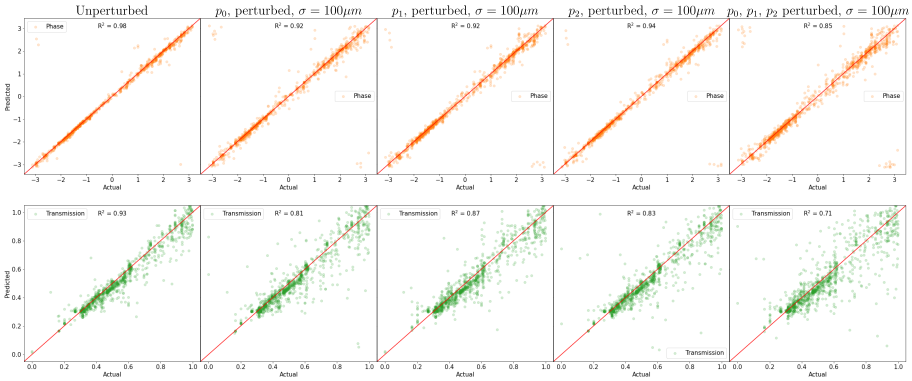

Fig. S8 Robustness Analysis of Scatterers. Each individual subplot represents a comparison between the unperturbed expected values (x-axis) and the predictions made by the meta-model (y-axis). The first row of subplots exhibits the phase modulation, while the second row is devoted to the transmission modulation. An R^2 value is depicted in each subplot to illustrate data correlation with the y=x line. The columns of subplots are representative of varying perturbation conditions, extending from the unperturbed state to a condition where all three scalar parameters (p_0, p_1, p_2) are perturbed. The standard deviation of the perturbation is denoted by σ.

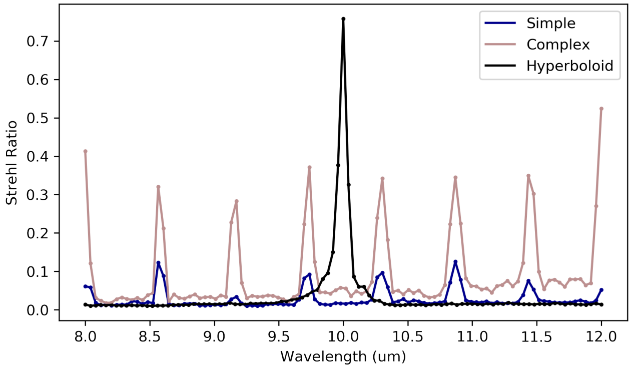

Fig. S9 Ideal Strehl Ratios from unperturbed scatterers.

S7 Fabrication Schematic

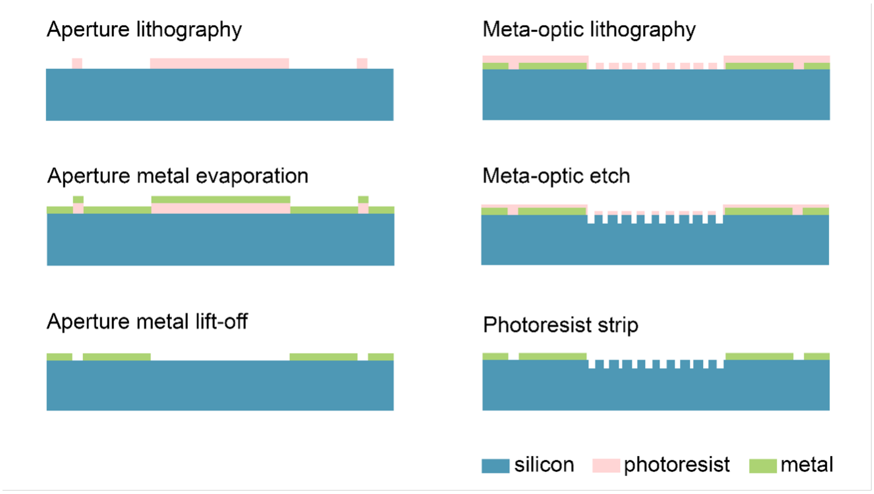

Fig. S10 Pictorial representation of fabrication process.

Supplementary References

[1] Zhan, A., Colburn, S., Trivedi, R., Fryett, T.K., Dodson, C.M., Majumdar, A.: Low-contrast dielectric metasurface optics. ACS Photonics **3**(2), 209–214 (2016)

[2] Colburn, S., Zhan, A., Bayati, E., Whitehead, J., Ryou, A., Huang, L., Majumdar, A.: Broadband transparent and cmos-compatible flat optics with silicon nitride metasurfaces. Optical Materials Express **8**(8), 2330–2344 (2018)
